# Vascularization Characteristics of the Different Meniscal Layers: Three-Dimensional Assessment With Micro-CT

**DOI:** 10.1177/23259671251341472

**Published:** 2025-06-09

**Authors:** Federica Orellana, Stefano Zaffagnini, Ruslan Hlushchuk, Oleksiy-Zakhar Khoma, Sébastien Halm, Annapaola Parrilli, Alberto Grassi

**Affiliations:** * Empa—Swiss Federal Laboratories for Materials Science and Technology, Center for X-ray Analytics, Dübendorf, Switzerland; † IRCCS—Rizzoli Orthopaedic Institute, 2nd Orthopaedic and Traumatologic Clinic, Bologna, Italy; ‡ University of Bern, Institute of Anatomy, Bern, Switzerland; Investigation performed at Empa, Dübendorf, Switzerland

**Keywords:** micro-CT, meniscus, meniscal vascular network, meniscal tears, 3D imaging

## Abstract

**Background:**

Degenerative meniscal lesions are often characterized by horizontal cleavage tears, and there is currently no established gold standard for treating these injuries. Understanding the vascularization and distribution of blood vessels along the meniscal layers could offer valuable insights into the management and healing of these tears.

**Hypothesis:**

Distinct vascularization patterns could be identified in different layers of the meniscus along the proximal-distal axis, providing new insights and potentially expanding or refining existing classification systems.

**Study Design:**

Descriptive laboratory study.

**Methods:**

To visualize the meniscal microvasculature, human cadaveric legs were perfused with a polymer-based contrast agent, followed by micro–computed tomography imaging. The menisci were virtually divided into transverse (layers), radial (thirds), and circumferential zones to quantify the vascular contribution of each zone and to evaluate vascular parameters such as segment diameter, tortuosity, and the number of vessel segments.

**Results:**

In the medial meniscus, the inferior surface of the outermost zone and the intermediate layers of the peripheral zones showed a potential reduction in vessel percentage. In the lateral meniscus, a relatively higher percentage of vessels was observed from the superficial to the inferior layers of the posterior horn, extending into the inner zones. Additionally, the lateral meniscus exhibited a greater number of vessels with smaller diameters compared with the medial meniscus.

**Conclusion:**

This micro-computed tomography approach for analyzing spatial vascular distribution offers a more comprehensive view of meniscal vasculature across multiple planes and regions.

**Clinical Relevance:**

This study may pave the way for new classifications that identify highly and poorly vascularized regions of the meniscus, potentially improving the effectiveness of treatments for meniscal injuries, ultimately reducing recovery time and improving long-term joint health for patients.

The unique vascular supply of the meniscus, first described by Arnoczky and Warren,^2^ varies with age and zone. This results in the classification of the meniscus into red-red, red-white, and white-white zones with decreasing blood supply. Although this type of nomenclature is still commonly used, the Meniscal Documentation Subcommittee of the International Society of Arthroscopy, Knee Surgery and Orthopaedic Sports Medicine (ISAKOS) has discouraged the use of this nomenclature for meniscal tears^1^ because it does not fully account for the variability in vascular supply across the meniscus. Nevertheless, understanding meniscal vessel distribution remains critical to determining appropriate treatments and healing potential.^14^

Degenerative meniscal tears are usually characterized by horizontal cleavage tears,^9^ with gross delamination between meniscal layers. This division of the tissue creates a superior and inferior leaflet that can extend through the articular or peripheral region of the meniscus. To date, the pathogenesis of this type of lesion is still not fully understood, and there is no gold-standard treatment among leaving it alone, complete resection, resection of the superior or inferior leaflet, and repair.^3^ The success of these repairs is not always predictable; some vascularized areas may fail to heal completely, while some nonvascularized areas may recover unexpectedly.^13,19^ A better understanding of the vascularization of the different meniscal layers and the distribution of vessels along the radial course could significantly improve the management of these tears. Furthermore, this insight highlights the importance of studying meniscal vascularization in 3 dimensions when considering healing and treatment strategies.

Given the above, it is hypothesized that new topographical divisions of the meniscal areas and potential new classifications could be beneficial. For instance, there may be implications for the proximal or distal zones of the meniscus along its axis (eg, layers) in combination with the circumferential and radial areas. This proposed reclassification could have significant implications, for example, in influencing surgical treatment such as the pattern of meniscal debridement or suturing techniques and the choice of suturing locations.

Specifically, suturing techniques vary based on meniscal anatomy and the tear location. Developing these new classifications, aimed at delineating areas with potentially less variability, could significantly enhance the outcomes of meniscal treatment by optimizing the preservation of the most vascularized part of the menisci and the healing environment and minimizing the risk of further injury.

Therefore, the purpose of our work was to provide initial insight into the behavior and quantification of vessels along the proximal-distal axis of the meniscus. This study aimed to fill a significant gap in the existing literature by providing detailed 3-dimensional (3D) methods for an in-depth analysis of the vascular distribution. We hypothesized that different patterns of vascularization could be found in different meniscal layers, in the proximal-distal axis.

## Methods

### Perfusion and Sample Preparation

All procedures were conducted in strict accordance with applicable guidelines and regulations. Three Thiel-fixated human cadaveric legs, comprising 6 menisci, were donated by male individuals with a mean age of 75 years (range, 62-86 years). Before perfusion, the polymer-based contrast agent µAngiofil (Fumedica; Muri AG) was prepared according to the manufacturer’s guidelines. Cannulation of the femoral artery of the corresponding limb was performed using a 14-gauge angiocatheter needle for perfusion. The limb was perfused with low-viscosity silicone oil (Bluesil; Elkem), supplemented with blue dye (Orasol Blue dye; BASF), to dislodge postmortem clots and restore flow to the region of interest. Perfusion with oil was continued until the skin near the knee joint turned blue, indicating successful perfusion of the microvascular bed around the knee. Subsequently, the previously prepared µAngiofil was injected through the cannulated femoral artery at a rate of 20 mL/min. After perfusion, the sample was kept in a vertical position for 30 minutes to allow complete polymerization of the contrast agent. The knee was then excised with a cut approximately 10 cm above and below the tibiofemoral joint. The medial and lateral menisci were carefully isolated from the knee joint capsule and cut at the level of the anterior and posterior roots. Whole menisci were fixed for 48 hours in 4% paraformaldehyde.

### Ethics Declarations

The use of the human cadaveric material was performed according to the Swiss federal act on research involving human beings (No. 810.30, Human Research Act of the Federal Assembly of the Swiss Confederation) of September 30, 2011, and the guidelines of the Swiss Academy of Medical Sciences, updated in 2014. Donors formally agreed to the use of their body parts for research by signing the informed consent forms. The experimental protocols have been approved by the Management Board of the Institute of Anatomy, University of Bern.

### Micro–Computed Tomography Imaging

Micro–computed tomography (micro-CT) analysis utilized an EasyTom XL Ultra 230-160 micro-/nano-CT scanner (RX Solutions). Image acquisition involved a rotation step of 0.25° and a frame average of 10. Operating parameters included 70 kV and 70 μA, with a nominal resolution of 30 μm. Reconstruction of all CT data sets used the filtered back-projection algorithm, along with small ring artifact reduction and application of a 65% Hann window function. The segmentation of the meniscal vascular network was achieved through a combination of the maximum entropy algorithm and the white top-hat operation as described in a previous article,^14^ utilizing the open-source image processing package Fiji^16^ in conjunction with the Avizo software application (Thermo Fisher Scientific).

This integrated segmentation process was used because the maximum entropy algorithm effectively extracts the primary vascular network by identifying the optimal grayscale range for larger, highly attenuating blood vessels, while the white top-hat method isolates finer details, including smaller vessels and brighter elements. The centerlines of the filamentous structures were extracted from the segmented image using the Auto Skeleton module (Avizo 3D)^6^ to analyze individual vessel branches.

The menisci were virtually divided into 3 transverse layers to evaluate these parameters along the proximal-distal axis, with each section representing one-third of the meniscus. These areas were designated as superior, intermediate, and inferior, and all parameters were calculated for the whole menisci in relation to the 3 transverse areas. Each transverse zone was then divided into 3 radial areas (anterior, middle, and posterior), each comprising one-third of the meniscus, and 4 circumferential zones defined by the ISAKOS zone classification system (zone 0: perimeniscal; zone 1: outer third; zone 2: middle third; zone 3: inner third).^1^ The meniscal vascular contribution for the layers in the circumferential and radial zones was determined as the ratio in percentage between the voxels identified as vessels for each zone and the total number of voxels associated with the vascular network of the corresponding layer. The vascular network was assessed by evaluating important vascular parameters, including segment diameter, tortuosity, and number of segments.

### Statistical Analysis

All data are presented as mean ± standard error of the mean. Single-factor analysis of variance and the Tukey-Kramer test were performed for multiple comparisons. Statistical significance was set at a *P* value <.05.

## Results

### 3D Imaging of the Meniscal Vascular Network

**Figure 1.**
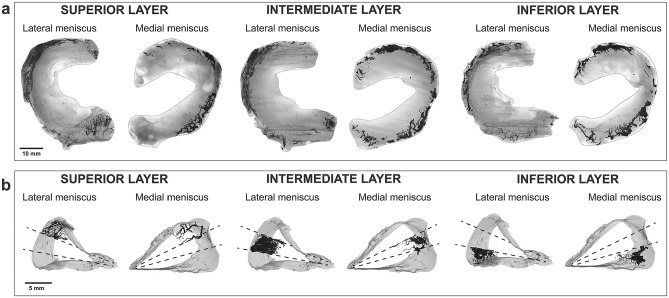
Vasculature of the human lateral and medial menisci visualized by micro–computed tomography. (a) Axial 3-dimensional view of the 3 transverse layers of the lateral and medial menisci. Scale bar, 10 mm. (b) Radial 3-dimensional view of the 3 transverse layers of the lateral and medial menisci. Scale bar, 5 mm.

The 3D morphology and architecture of the medial and lateral menisci, along with their respective vascular networks across the superior, intermediate, and inferior layers, are illustrated in Figure 1. The 3D imaging showed that no meniscal layer is completely devoid of blood vessels. Indeed, the vascular network similarly supplies the superior, intermediate, and inferior layers of the meniscal tissue. The radial 3D views of the meniscus (Figure 1b) allowed a visual depiction of the circumferential and radial vessel branches that penetrate the meniscus.

### Meniscal Vascular Volume Contribution in the Respective Layers and Circumferential Zones

In both the lateral and medial menisci, the vascular contribution of the transverse layers varies across different circumferential zones.

#### Lateral Meniscus

**Figure 2.**
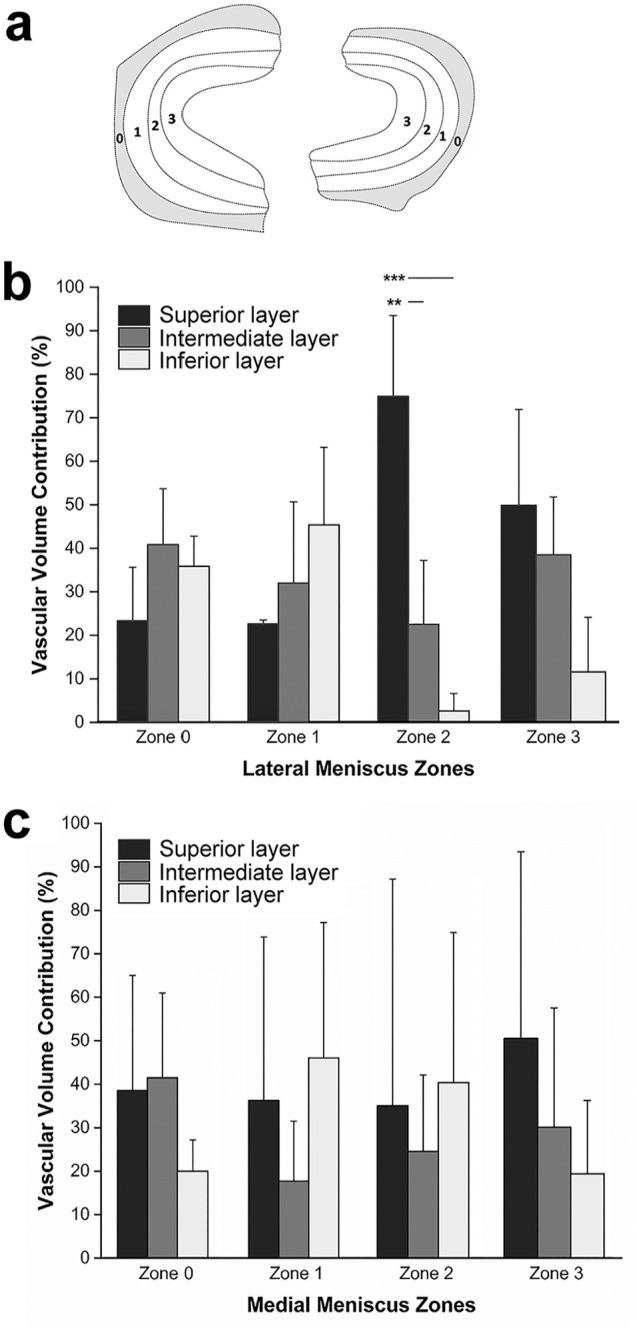
Meniscal vascular volume contribution in the respective layers and circumferential zones. (a) Schematic representation of the circumferential zones of both the lateral and medial menisci. (b) Vascular volume contribution in the lateral meniscus (n = 3 for each zone). (c) Vascular volume contribution in the medial meniscus (n = 3 for each zone).

In circumferential zones 0 and 1, the superior layer is the least vascularized (23% for each circumferential zone). In contrast, the intermediate layer (41%) and the inferior layer (45%) are the most vascularized for zones 0 and 1, respectively. In the inner circumferential zones of the tissue (zones 2 and 3), blood vessels show a similar pattern, with more vascular volume in the superior layer (zone 2: 75%; zone 3: 50%) and less in the inferior layer (zone 2: 3%; zone 3: 12%) (Figure 2b).

#### Medial Meniscus

The inferior layer is the least vascularized in circumferential zones 0 and 3 (zone 0: 20%; zone 3: 19%). In zone 0, the superior and intermediate layers have a very similar quantity of blood vessels, with 39% and 41%, respectively. In circumferential zones 1 and 2, a similar trend in the vascular contribution of the 3 transverse layers can be observed. In both zones, the intermediate layer shows the least blood vessel volume (zone 1: 18%; zone 2: 25%). In zone 3, the superior layer contains more than half of the vascular network of that area (51%), while the intermediate layer contains 30% of blood vessels (Figure 2c).

### Meniscal Vascular Volume Contribution in the Respective Layers and Radial Zones

Similarly to the circumferential zones, the radial zones also have different distributions of blood vessels in the various transverse layers.

#### Lateral Meniscus

**Figure 3.**
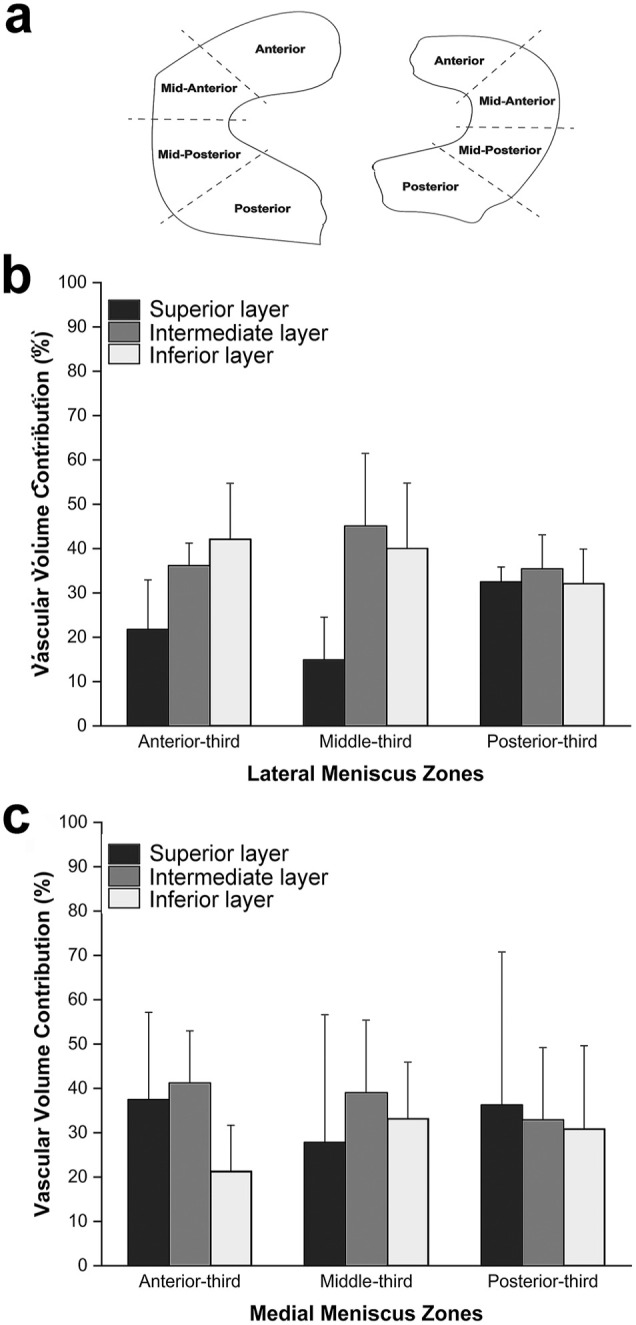
Meniscal vascular volume contribution in the respective layers and radial zones. (a) Schematic representation of the radial zones of both the lateral and medial menisci. (b) Vascular volume contribution in the lateral meniscus (n = 3 for each transverse zone). (c) Vascular volume contribution in the medial meniscus (n = 3 for each transverse zone).

The most vascularized areas are the inferior layer in the anterior third (42%) and the intermediate layer in the middle (45%) and posterior third (35%) (Figure 3b). The least vascularized areas are the superior layer in the anterior-third and middle-third regions, with 22% and 15%, respectively, of the total vascular volume of the corresponding radial area. The superior and inferior layers of the posterior portion of the lateral meniscus are equally vascularized (32%-33%) (Figure 3b).

#### Medial Meniscus

The least vascularized areas are the inferior layer in the anterior portion (21%) and the superior layer in the middle portion (28%) (Figure 3c). The posterior layer of the medial meniscus shows similar vascularization levels across all 3 transverse layers: 36% for the superior layer, 33% for the intermediate layer, and 31% for the inferior layer (Figure 3c).

### Vascular Topology and Connectivity

#### Lateral Meniscus

The intermediate layer results from having a vascular network with a greater volume and greater number of segments than the superior and inferior layers. Additionally, the vascular segments of the intermediate layer are distinguished by having a greater diameter in comparison with the other 2 transverse layers of the tissue. The superior layer has the lowest number of vascular segments, forming a vascular network with a smaller total volume compared with the intermediate and inferior layers. Still in the superior layer, the mean diameter of the blood vessels is statistically smaller than in the intermediate and inferior layers (superior vs intermediate layers: *P* = .0103; superior vs inferior layers: *P* < .00001).

#### Medial Meniscus

Similarly to the lateral meniscus, the intermediate layer of the medial meniscus possesses a vascular network with a greater volume and mean diameter. This vascular network, however, does not contain the greatest number of vascular segments, a parameter that is greater in the inferior layer of the tissue. The mean diameter value is different in all 3 transverse layers in a statistically significant way, resulting in a smaller value in the inferior layer and a bigger value in the intermediate layer (superior vs intermediate layers: *P* < .00001; superior vs inferior layers: *P* = .00004; intermediate vs inferior layers: *P* < .00001).

**Figure 4.**
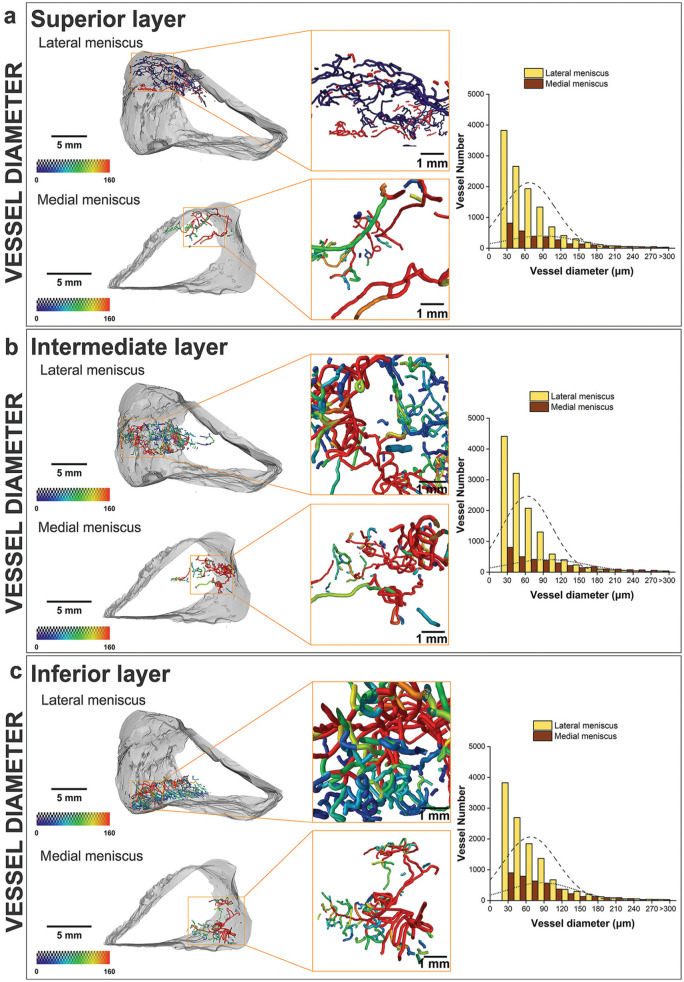
Visualization of vessel diameter in the human lateral and medial menisci using micro–computed tomography. Radial 3-dimensional views and vessel diameter distribution of the (a) superior, (b) intermediate, and (c) inferior transverse layers. Zoomed areas are displayed on the right-hand side of each radial portion. Blood vessels are color-coded according to their diameter (range, 0-160 μm). In the vessel diameter distribution panel, dashed and dotted lines indicate the mean frequencies of the vessel diameters for the lateral and medial menisci, respectively. Scale bars, 5 mm (radial portions) and 1 mm (zoomed-in areas).

The blood segments forming the vascular network of each transverse layer were visualized and characterized based on their diameter (Figure 4). The vascular segments within all transverse layers along the proximal-distal axis of both the lateral and medial menisci exhibit a comparable range of diameter values, distinguishable by blood vessel coloring ranging from blue to red (0-160 μm). However, the lateral meniscus contains a greater total number of vessels, including a greater proportion of smaller vessels, compared with the medial meniscus, in all 3 transverse layers. This trend is also visible in the 3D volume renderings, where the superior layer of the lateral meniscus displays smaller-diameter blood vessels, discernible by their purple coloration (Figure 4).

## Discussion

The most important finding of the present study was that through the 3D micro-CT protocol, it was possible to identify different vascularization patterns in meniscal layers. This study serves as a proof of concept and methodological proposal, providing initial insight into the 3D morphology and quantification of vessels along the proximal-distal axis of the meniscus. By focusing on key parameters such as volume, vessel diameter, and tortuosity, we aimed to establish a basic understanding of the vascular network within the meniscus and, in particular, along the proximal-distal axis and layers. This understanding may lead to new classifications that better identify the most favorably vascularized and nonvascularized areas of the meniscus, ultimately improving the diagnosis and treatment of meniscal lesions. By incorporating our approach, we could refine meniscal repair techniques, resulting in more predictable outcomes and improved healing. This would mark a significant advancement in effectively managing meniscal injuries.

### General Considerations

Several studies have indicated that the vascular supply and healing potential of the meniscus vary significantly, depending on its location. However, to our knowledge, no other studies have analyzed the transverse sections and the layers along the proximal-distal axis. The meniscus is known to have a peripheral vascular supply that diminishes toward the central regions, influencing its healing capacity.^2,15^ However, the variability and the limited detailed 3D information on the vascularization and its parameters do not allow a clear classification into well-defined zones, despite the observed trends. Our idea of adding a new perspective to the traditional circumferential and radial^5^ subdivisions aims to potentially provide a more precise anatomic background for clinicians in devising surgical strategies depending on the type of lesion and localization. In addition, our approach seeks to enrich the traditional classifications by integrating 3D vascular parameters that may better reflect the functional and structural intricacies of the meniscus. For example, understanding the precise 3D vascular patterns may guide the technique of meniscal resection or repair, and the determination of optimal repair sites, potentially improving surgical success rates.

Despite the promise of our approach, especially as the first of its kind, it is important to recognize the limitations of the present study. In particular, the small sample size and the advanced age of the specimens, in which degenerative processes may have affected the actual vascularization, could significantly limit the generalizability of our findings. Given the lack of existing references, future studies with larger cohorts and specimens with younger age are essential to validate our preliminary observations and to develop robust, clinically applicable guidelines.

### Lateral Meniscus

Our results reveal important variations in vascular contribution across different circumferential zones along the proximal-distal axis of both the lateral and medial menisci. In the lateral meniscus, a similar vascularization across all the layers was noted in the posterior segment, indicating a possible higher healing potential. Interestingly, the vascularization of the superior layer reached 75% of the volume in zone 2, which is an area close to the free margin that is considered less vascularized.^14^ This could be responsible for the spontaneous healing of small partial tears and the high rate of success of surgical repair of complex lateral meniscus oblique radial tears or root tears.^8^ Based on these findings, surgical repair in the posterior horn should be encouraged, with no adjunct of biological augmentation. In contrast, the midbody and anterior segment exhibited a vascular reduction in the superior layers, which could be related to the free margin of the superior border of the meniscus corresponding to the popliteal hiatus. This raises critical issues in healing of the tear in this location, especially for radial tears.^4,10^

Another interesting finding is the higher density of small-diameter vessels in all 3 transverse layers of the lateral meniscus compared with the medial meniscus, especially in the superior one. This could indicate a significant capacity for nutrient delivery and waste removal, thus potentially enhancing the healing potential and recovery from microtraumatic events despite the lower overall vascular volume.

### Medial Meniscus

For the medial meniscus, the vascular distribution also varies notably. In this case, in the more vascularized perimeniscal zone (zone 0), the superior and intermediate areas have a comparable percentage of blood vessel contribution (39% and 41%, respectively), while the inferior area close to the tibia has a lower percentage (20%). The inferior surface of the posterior horn is the typical location of hidden ramp lesions.^17,18^ The peculiar vascularization of this area could indicate a critical zone for meniscal tears. A careful arthroscopic inspection is recommended because overlooking a lesion in this area could result in tear progression, if not properly treated. Surgical repair is advised and could also be considered in stable tears.

When the radial distribution of vessels is considered, a low percentage of vessels is reported in the intermediate layer in zones 1, 2, and 3. Alternatively, the inferior layer demonstrated a progressive decrease from zone 1 to zone 3, and the superior layer exhibited the higher vessel distribution, which was stable along all the meniscal radius. This peculiar vessel distribution could identify the intermediate zone as the “weak ring” of the medial meniscus from the biological point of view and could explain the genesis of degenerative horizontal cleavage tears in this location. For this reason, a biological augmentation could be considered when attempting repair in this area, possibly after debriding the less vascular outer margin (zone 3).^7^ Conversely, in the case of isolated debridement of a single leaflet without meniscal repair, the data on vascularization reported in this study suggest removal of the inferior meniscal portion, which presents a lower percentage of vessels, especially in the anterior third.

Finally, the medial meniscus presented an overall lower number of vessels, which exhibited a higher diameter compared with the ones in the lateral meniscus. This specific “biological” difference,^11^ together with the different biomechanical behavior,^12^ could be responsible for the lower healing potential of the medial meniscus and higher failure rate of surgical repair in the medial side.^13^

The findings and observations from this study could lead to the development of new classification systems that more accurately identify highly and poorly vascularized regions of the meniscus, potentially increasing the effectiveness of treatments for meniscal injuries, reducing recovery times, and improving long-term joint health for patients. Further research, building on this landmark study, is needed to explore the functional implications of these findings and to refine surgical interventions based on this new knowledge.

## Conclusion

The present 3D micro-CT method to analyze vascular distribution provided a more comprehensive view of the meniscal vasculature in multiple planes and different areas. In the medial meniscus, the inferior surface of the most peripheral zone and the intermediate layers of the outer zones were identified as potential sites of decreased percentage of vessels. In the lateral meniscus, a relatively high percentage of vessels was identified from the superficial to the inferior layers in the posterior horn, up to the inner zones. Moreover, a higher absolute number of vessels but with a smaller diameter were identified in the lateral meniscus with respect to the medial one.
